# Using brain functional magnetic resonance imaging to evaluate the effectiveness of acupuncture combined with mirror therapy on upper limb function in patients with cerebral ischemic stroke: a study protocol for a randomized, controlled trial

**DOI:** 10.1186/s13063-020-04955-2

**Published:** 2021-01-12

**Authors:** Mingzhu Xu, Run Lin, Jing Luo, Chunzhi Tang, Shuhui Wang, John Wong, Meng Wu, Jianting Huang, Peng Shi, Ang Gao, Yuqian Bai, Ying Xie, Jun Luo, Yunqiu Yang, Shaoyang Cui

**Affiliations:** 1 Shenzhen Hospital of Guangzhou University of Chinese Medicine (Futian), Shenzhen, 518034 Guangdong China; 2 Shenzhen Hospital, Southern Medical University, Shenzhen, 518100 Guangdong China; 3 Shenzhen Hospital, Peking University, Shenzhen, 518034 Guangdong China; 4 Guangzhou University of Chinese Medicine, Guangzhou, 510405 China; 5 MGH Institute of Health Professions, Boston, MA USA; 6 Department of Biomedical Engineering, City University of Hong Kong, Kowloon Tong, Hong Kong; 7 Shenzhen Institutes of Advanced Technology, Chinese Academy of Sciences, Beijing, China; 8 Shenzhen Prevention and Treatment Center for Occupational Diseases, Shenzhen, 518001 China; 9 Macau University of Science and Technology, Macau, 519020 China

**Keywords:** Upper limb and hand motor dysfunction, Cerebral ischemia, Mirror therapy, Jin’s three-needle acupuncture, fMRI, Randomized controlled trial

## Abstract

**Background:**

Upper limb and hand motor dysfunction is one of the challenges in rehabilitation after cerebral ischemic stroke (CIS), and the clinical efficacy of rehabilitation needs to be improved. This study aims to combine Jin’s three-needle acupuncture (JTN) therapy with mirror therapy (MT) for hemiplegia after CIS, objectively evaluate the clinical effects and safety of JTN to treat upper limb dysfunction, and use functional magnetic resonance imaging (fMRI) of the brain to investigate the central mechanisms of the effects, which would provide a powerful evidence-based medical basis for further supporting the application of JTN combined with MT.

**Methods/design:**

This trial will be a single-blind, randomized controlled study. Patients who meet the study criteria will be recruited and randomly assigned to either the combined treatment group (JTN+MT) or the JTN group. Both interventions will be conducted for 6 days per week and last for 4 weeks. The primary outcome will be the effective rate based on the Fugl–Meyer Assessment for Upper Extremity (FMA-UE). Other outcome measures will include scores on the motor assessment scale (MAS), action research arm test (ARAT), activities of daily living (ADL) scale, and fMRI analyses. For safety evaluation, adverse events will be observed and recorded.

**Discussion:**

This study may help to identify the efficacy and safety of acupuncture combined with MT for upper limb dysfunction after CIS and explore the central mechanisms with brain fMRI.

**Trial registration:**

Chinese Clinical Trial Registry ChiCTR-IOR-17012174. Registered on 5 April 2017.

## Background

Cerebral ischemic stroke (CIS) accounts for 80% of 12.42 million stroke patients aged 40 or older in China in 2017 [1]. If not promptly and adequately diagnosed and treated, CIS patients will suffer serious damage to the nervous system, become handicapped, or even die because of infectious complications [2].

Upper limb motor impairment is a common sequelae after stroke and restricts function in muscle movement or mobility [3], manifested as coordination and execution problems of the arms, palms, and fingers that result in restrictions on daily activities such as eating, dressing, and bathing [4]. Eighty percent of patients experience acute paresis of the upper extremity after stroke, and about 37% of the patients still have problems such as decreased control of upper limbs and hand fine motor skills [5] 3 months after stroke onset. However, rehabilitation of CIS patients with upper limb motor impairment continues to be challenging. We propose combining the use of Western Medicine and Traditional Chinese Medicine and determining whether the combination therapy will provide improved outcomes.

Functional magnetic resonance imaging (fMRI) technique, a noninvasive imaging technique with exceptional spatial and temporal resolution, is frequently used to study brain functional connectivities [6] and can monitor the progress of cortical functional remolding in stroke patients [7–9]. Because it can accurately determine whether the brain function in the motor areas disappears or shifts and whether there is reconstruction of the brain functional areas around the lesion [10], the technique can guide clinical rehabilitation and prognosis of patients.

Acupuncture has been used as part of Traditional Chinese Medicine (TCM) therapy to treat stroke for thousands of years. Jin’s three-needle acupuncture (JTN) therapy is a popular acupuncture technique due to its simple and easy manipulation. First developed by Rui Jin, a professor of Guangzhou University of Chinese Medicine, it has been widely used in China to treat post-stroke hemiplegia in the past decades. Satisfactory and promising effects have been reported in clinical trials [11–13].

Mirror therapy (MT), also known as mirror visual feedback (MVF) therapy [14], is a treatment method based on visual illusion, visual feedback, and virtual reality combined with a rehabilitation training program [5]. Previous studies have shown that MT was significantly associated with immediate improvement of upper extremity motor function in stroke patients [6, 15, 16] and has gradually been accepted by clinical researchers as a new treatment for post-stroke limb rehabilitation [6, 17].

Therefore, we hypothesize that the clinical efficacy of CIS will be improved with the combination of acupuncture therapy and MT. Based on the modern image technique, we may also find out the central brain mechanisms which could provide a strong evidence-based medical basis for further popularizing the application of JTN+MT in the treatment on upper limb function in patients with cerebral ischemic stroke.

## Methods/design

### Study design

**Fig. 1:**
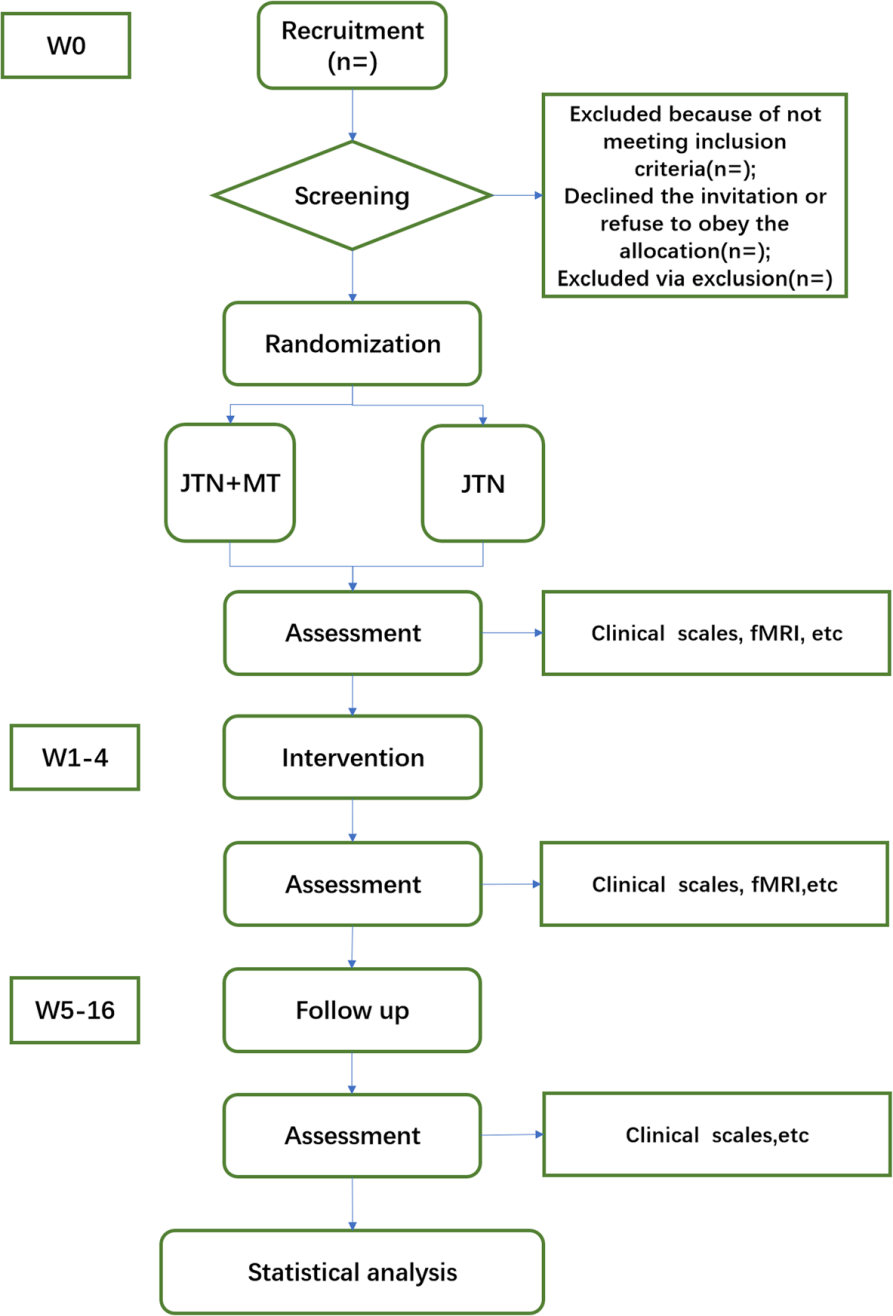
Flowchart of the trial. W, week, JTN, Jin’s three-needle acupuncture, MT, mirror therapy, fMRI, functional magnetic resonance imaging

This is a single-blind randomized controlled trial. The study objective is to explore the central brain mechanisms of JTN combined with MT to relieve carotid atherosclerotic plaques by establishing JTN+MT and JTN groups. Two groups will be randomly established at a 1:1 ratio, including the JTN+MT group (receiving Jin’s three-needle acupuncture therapy and mirror therapy) and the JIN group (receiving Jin’s three-needle acupuncture therapy). The study will enroll 60 individuals in total, 30 individuals in each group. JIN group is set to evaluate the clinical efficacy of JIN for CIS. The flowchart of this protocol can be found in Fig. 1.

### Inclusion criteria

The inclusion criteria include the following: (1) corresponding to the diagnostic standards of CIS [18]; (2) corresponding to the diagnostic standards of stroke of TCM [19]; (3) diagnosed with CIS and confirmed by CT or MRI; (4) first-ever clinical CIS occurred ≥ 2 weeks and ≤ 6 months previously, the number of times ≤ 2; (5) aged between 40 and 75, male or female; (6) limb function in NDS [20] score ≥ 10; (7) stable vital sign and consciousness; (8) normal test result of kinesthetic and imagery questionnaire [21]; and (9) provided written informed consent to participate.

### Exclusion criteria [7]

The exclusion criteria include the following: (1) transient ischemic attack (TIA); (2) upper limb dysfunction caused by brain tumor, brain trauma, and other diseases; (3) craniotomy requirement or hematencephalon; (4) sever spastic deformation on upper limb; (5) pregnant and lactating women; and (6) severe complications of heart, liver, kidney, hematopoietic system, and endocrine system or with a severe mental illness that prevents them from cooperating with treatment, including addiction and substance use disorders, dissociative disorders, and schizophrenia.

### Study setting

**Fig. 2:**
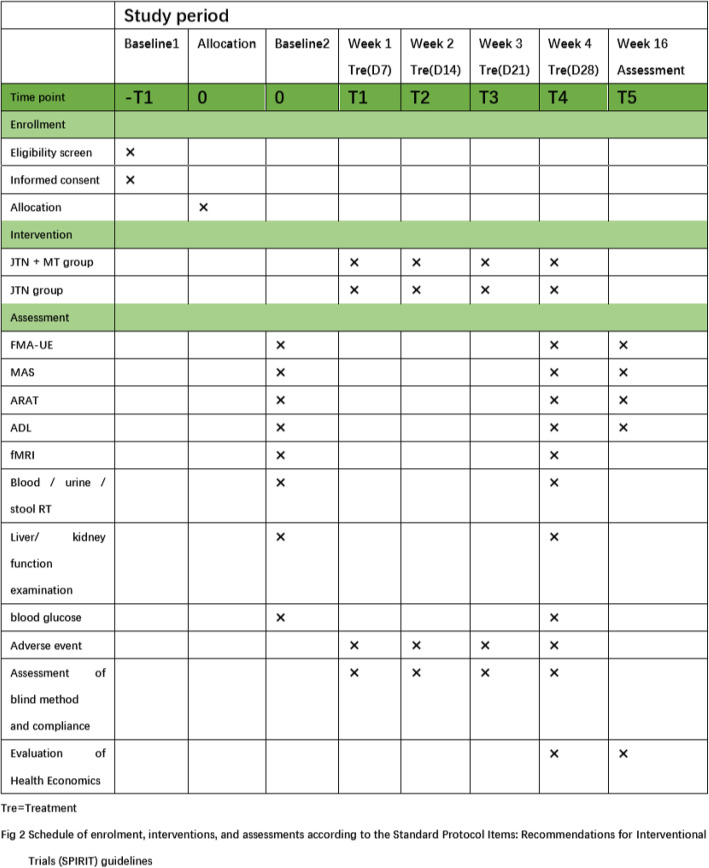
Schedule of enrolment, interventions, and assessments according to the Standard Protocol Items: Recommendations for Interventional Trials (SPIRIT) guidelines. D, day; T, time; Tre, treatment; JTN, Jin’s three-needle acupuncture; MT, mirror therapy; FMA-UE, Fugl–Meyer Assessment for Upper Extremity; MAS, motor assessment scale; ARAT, the action research arm test; ADL, activities of daily living; fMRI, functional magnetic resonance imaging; RT, routine test

The treatment will be conducted at the rehabilitation department of Shenzhen Hospital of Guangzhou University of Traditional Chinese Medicine (Futian). The study will enroll 60 individuals in total, with 30 individuals in each group. Patients who are included in the study are required to sign an informed consent form and will participate in the study on a voluntary basis. The researchers will obtain written informed consent from each participant before screening. The informed consent form (in Chinese) is provided in Supplemental file 2. The schedule of patient enrolment, intervention, and assessment is illustrated in Fig. 2.

### Participants

#### Recruitment procedures

Researchers of the study will recruit 60 eligible patients with upper limb motor dysfunction after CIS from Shenzhen Hospital of Guangzhou University of Traditional Chinese Medicine (Futian). Eligible patients must meet the inclusion and exclusion criteria. Information about this trial will be available through posters, and leaflets will be distributed to inpatients and outpatients at multiple locations in the hospital. Interested patients can contact the project leader through their therapists, among others, by telephone and email. If an applicant meets the study criteria, they will be invited to participate. The following basic personal information will be collected: sex, age, body mass index (BMI), educational background, occupation, marital status, related past health history, etc. These details about the participants will be maintained by the data monitoring committee (DMC) and will never be revealed to any individual or organization not connected to the study.

#### Randomization

A total of 60 participants who meet the inclusion criteria will be randomly allocated into the combined treatment group (JTN+MT) and JTN group. A statistician blinded to the treatment and data collection will use Strategic Applications Software (SAS, version 9.1.3, SAS Institute Inc., Cary, NC, USA) to generate the random allocation sequence. The group name will be written on a card and sealed in an opaque envelope. The sequence numbers will be written on the envelopes, and the envelopes will be numbered sequentially. Random allocation will be performed for participants who meet all selection standards and signed the Consent Form. The research coordinator will allot participant identification codes and record the codes in the Case Report Forms. The therapists will open random allocation envelopes sequentially and allocate the participants accordingly. The details (such as name, sex, age, and date of inclusion) of newly included participants will be recorded before randomization. The opened envelopes will be separately stored in lockers.

#### Blinding

Because of the characteristics of therapy, the acupuncturists in this study cannot be blinded to the assignment while the participants cannot be blinded to the MT, but the outcome assessors, data manager, and statistics analyzer will evaluate the results of the study without being aware of subject allocation. The evaluators of the final intervention effect are blinded that can effectively avoid the bias caused by subjective factors in the evaluation. The blinding will be monitored and assessed by independent statisticians responsible for data monitoring and by the Ethics Committee of the study center. The blind code will be disclosed after completion of the statistical analysis.

### Intervention

Both treatments will be conducted for six consecutive days per week and last for 4 weeks. We will use No. 30 disposable acupuncture needles (size 0.32 mm × 25 mm or 0.32 mm × 50 mm stainless steel disposable acupuncture needles (Suzhou Huatuo Medical Instrument Factory) for the intervention. The quality of the needles will be checked before conducting the treatment to ensure safety.

The practitioners of this trial have more than 5 years of clinical experience and have participated in a training session to ensure that the treatments will be administered consistently. The patient may make an appointment with the physician before starting the treatment, and confirmation will be made by a phone call to enhance compliance.

#### JTN combined with MT group

This study uses the method of staged acupuncture [22]. According to Jin’s three-needle theory combined with previous related research [23], every point group is compatible with some kind of disease or a series of symptoms. Thus, in the soft paralysis period, temporal three needles [temporal I needle: 2 cun (a unit of length (=1/3 decimeter)) on the tip of the ear; temporal II and temporal III needle: 1 cun in front of and behind the temporal I needle] and hand three needles [LI11, TE5, LI4] will be used at the main point. In the spasm phase, temporal three needles and clonic three needles of the upper limbs [PC6, HT1, LU5] will be taken as the main point. Wisdom three needles [GV24, GB13] will be used with syndrome differentiation.

“Scalp-cortex theory” [24] has indicated that the effective rate of temporal acupuncture for stroke patients was 97%, among which 67.6% patients had significantly improved upper limb muscle strength. It has been reported [7] that hand three needles and clonic three needles of the upper limbs will coordinate and balance the muscle tension between agonistic muscles and antagonistic muscles, promote the transformation of joint movement to separate movement, and relieve upper limb tightness, paralysis, pain, sensory disturbance, and so on. The combined effect of these acupoints will promote the recovery of upper limb function.

Participants will be treated by therapists with more than 5 years of clinical experience. Patients will be in the supine position. Acupoints will be sterilized with alcohol, and the target position will then be fixed by the therapist’s left hand. The therapist’s right hand will insert thin, disposable acupuncture needles.

Among the selected points, LI11, TE5, LI4, PC6, HT1, and LU5 will be needled unilaterally based on the affected side of the patient. HT1 will be needled at 45°–90° to the body surface and inserted to a depth of 0.3–0.5 cun. TE5, LI4, and PC6 will be needled at 90° to the body surface and inserted to a depth of 0.5–1.0 cun. LI11 and LU5 will be needled at 90° to the body surface and inserted to a depth of 1.0–1.5 cun. Temporal three needles will be needled at 15°–30° to the body surface and inserted to a depth of 0.3–0.5 cun. Following insertion, stimulation of the acupuncture point will be performed with bidirectional rotation of the needle sleeve at approximately 18–300 per min. The acupuncture manipulation will be adjusted according to the tolerance of each patient with the methods of tonifying and purging to achieve the feeling of “deqi” without obvious pain. The needles will be maintained in place for 30 min. A sterilized, dry cotton ball will be gently pressed against the point to prevent bleeding when each needle is withdrawn. The entire course of treatment will occur over 30 min, while the upper limbs will be treated with MT.

During MT, patients who pass the kinesthetic and visual imagery questionnaire (KVIQ) test will sit close to the table. A 5 cm × 35 cm mirror will be placed vertically between the upper limbs on the table, with the reflecting surface facing the uninjured limb. Patients will be asked to observe the motion of the upper limb on the uninjured side and imagine that the limb on the affected side was in motion, imagine the motion of the affected limb the same as that observed on the uninjured side, and complete 6 movements including shoulder joint forward flexion, elbow joint flexion and extension, forearm forward and backward rotation, wrist joint flexion and extension, finger extension and grasping, and thumb abduction. The participants will be asked to perform each movement for 5 min and try to reach the maximum range of motion of the joints. Training will be completed for 30 min per day.

For the period of treatment, acupuncture and mirror training were performed 6 times per week for 4 weeks.

#### JTN group

In the control group, the acupuncture therapy will be the same as the treatment group’s.

#### Concomitant treatments

The patients in two groups will be treated with basic medications in the meantime. The patients in two groups will be treated with basic medications according to guidelines for clinical management of cerebrovascular disorders [25] to control blood pressure: individual treatment is adopted to stabilize blood pressure below 135/85 mmHg or within the normal range, control blood sugar and lipid, and prevent platelet aggregation.

The patients in the two groups will be provided with instructions to discourage them from receiving any additional specific complementary treatments related to upper limb dysfunction after CI throughout the trial, including abdominal acupuncture, fire acupuncture, floating acupuncture, and other acupuncture therapies, moxibustion, herbal medicine, massage, etc. If a patient needs other prescription medication, the relevant information will be recorded in the Case Report Form for that patient. The data will be assessed by two blinded researchers, to ensure whether be included.

For follow-up, all participants will participate in a 12-week follow-up after 4 weeks intervention. At the end of a 12-week follow-up period, participants will be referred for clinical evaluation to assess their functional status, including Fugl–Meyer Assessment for Upper Extremity (FMA-UE), motor assessment scale (MAS), action research arm test (ARAT), and activities of daily living (ADL) scale.

### Outcome measures

The study period is 16 weeks. FMA-UE, MAS, ARAT, ADL, and fMRI will be assessed before and after the treatment, as well as safety indicators such as blood routine test, urine routine test, stool routine test, liver and kidney function examination, and blood glucose. All the patients will take FMA-UE, MAS, ARAT, and ADL assesses after 12 weeks follow-up. Health economic indicators were recorded at week 4 and week 16. All outcomes will be measured by several experienced assessors who are blinded to the randomization group after the baseline visit for evaluation. All assessors will be trained to administer these assessments.

#### Primary outcomes

The primary outcomes in this study are the mean change in the scores on the FMA-UE from baseline to 4 weeks intervention and 12 weeks follow-up, as well as fMRI between the two groups and within groups from baseline to 4 weeks intervention.

FMA-UE is widely used for motor function assessment and can reflect the functional level of stroke subjects. The upper extremity has a maximum score of 66 points divided into three parts, namely shoulder arm (36 points), wrist hand (24 points), and coordination (6 points) and higher score indicate better upper limb movement function [4]. The FMA-UE is the most common scale for evaluating upper limb motor function after CIS. It is often used as a gold standard for testing the validity of other scales as well [26, 27]. The FMA-UE has excellent reliability and validity and is sensitive enough for clinical and research practice [28].

**Fig. 3:**
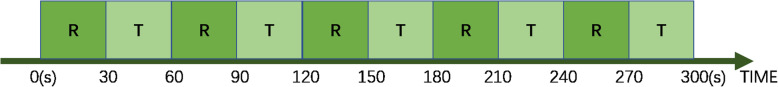
Task flowchart. R, rest; T, task

fMRI is a noninterventional imaging technique that uses magnetic resonance to measure changes in brain hemodynamics caused by neuronal activity as a reflection of the activation state of brain areas. For the observation methods, after the patients are ready, scanning will be performed on the whole brain of the patients. During resting-state scanning, patients will be instructed to relax, close their eyes, and avoid systematic thinking activities as much as possible. After resting-state scanning for 6 min, functional state scanning will be performed. The experimental task will be designed in blocks. There will be 10 trials for each task state (Fig. 3), and the time of each trial is 30 s. In addition, there are 10-s empty sweeps before each scan, so each scan is 5 min and 10 s. Once in place, the subjects will be put on noise-canceling earphones and receive instructions. Patients will be asked to try to imagine their fingers flexing when they hear the word “close.” When they hear the word “open,” they will be asked to try to imagine their fingers straightening. Each subject will receive task training in advance.

#### Secondary outcomes

The mean changes in the scores on the MAS, ARAT, and ADL before and after 4 weeks treatment as well as 12 weeks follow-up intervention are secondary outcomes.

MAS scale mainly focuses on comprehensive physical ability and muscle tension. It includes 9 items, from supine to the lateral position, from supine to sitting on the bed, seat balance, from seat to standing on foot, walking, upper limb function, hand movement, fine function of the hand, and muscle tension of the whole body. There are 7 points (0~6) for each of 9 items. Movement disorders can be divided into mild (33 points above), moderate (17~32 points), and severe (0 to 16 points) [29].

The ARAT is a standardized scale for the assessment of upper limb dysfunction after stroke. It evaluates the upper limb movement through four basic movements: grasping (6 items), gripping (4 items), pinching (6 items), and gross movements (3 items). Each project is on a 4-point scale, with 0 indicating failure and 3 indicating normal completion. Full score is 57 [4].

ADL scale includes 10 items related to bowel and bladder continence, grooming, toileting, feeding, transfer, walking, dressing, bathing, and going up and down stairs. A normal score is 100. Ability of daily life disorders can be divided into mild (61~99 points), moderate (41~60 points), severe (1~40 points), and completely dependent (0 points) [4].

### Sample size

The sample size was calculated based on the effective rate of the treatment. Based on similar previous studies [30], we assumed the effective rate of 88.9% and 56.1% in the JTN+MT group and the control group, respectively. The PASS11.0 software was used to perform a power analysis to determine the sample size. Type-1 error was assumed at 0.05, type-2 error at 0.2. The required sample size was 26 subjects for each group. Considering a dropout rate of 15%, a total of 60 subjects needed to be enrolled (30 participants per group).

### Adverse events and safety monitoring

Adverse events will be recorded and managed by researchers within 24 h. There will be at least one physician to evaluate and manage the adverse events at each center. Serious adverse and unexpected events will be reported to the Ethics Committee and other supervising departments. The subjects may leave the trial at their own discretion, or the physicians will determine whether the patient will continue or terminate the study. However, the patient will be followed up until they are in a stable condition following the adverse event.

The primary investigator will review all adverse events periodically, and the Ethics Committee and DMC will have access to the interim results. If necessary, a meeting will be held to reappraise the benefits and risk of this trial.

The possible adverse events related to acupuncture treatment include fainting during treatment, nausea, needle stuck, needle breakage, local hematoma, local infection, and viscera or peripheral nerve injury. Adverse events will be assessed based on the symptoms, frequency, and severity of the patients throughout the study. Patients who are intolerant to the treatment should be removed immediately from the trial.

### Data collection and statistical analysis

Therapists will collect the data using Case Report Forms. Two independent researchers will then input the data into the database. All analyses will be carried out under the guidance of independent data monitors to ensure the security and dependability of the data. About the dropped-out participants, their number and the reason for termination will be recorded, especially for adverse events. All enrolled patients will be included in the primary analysis conducted in accordance with the intention-to-treat (ITT) principle. The data will be analyzed by SPSS for Windows version 19.0. The statistical significance is defined as a two-sided *P* value of ≤ 0.05. The baseline characteristics will be reported as the means ± standard deviation (SD). For comparison of baseline materials, a chi-squared or Fisher’s exact test will be used for categorical variables, Student’s *t* test will be used for normally distributed data, and the Mann-Whitney *U* test will be used for non-normally distributed data. The scores of each rating scale and laboratory index before the intervention will be compared between the groups via Student’s *t* test or the Mann-Whitney *U* test. For the comparison within groups, the changes in the scores and indexes from baseline to endpoint will be assessed by a paired *t* test or the Wilcoxon rank-sum test. For the difference between groups, we will use Student’s *t* test or the Mann-Whitney *U* test. For the safety analysis, the adverse events will be listed and analyzed using a chi-squared or Fisher’s exact test. For participants who discontinue or deviate from intervention, listwise deletion or multiple imputation will be used.

The data of fMRI will be analyzed by professional technicians at the Shenzhen Advanced Institute.

### Ethics and dissemination

The study was planned in accordance with the Declaration of Helsinki. This trial protocol has been approved by the Ethics Committee of Shenzhen Hospital of Guangzhou University of Traditional Chinese Medicine (Futian) (2016-16) and registered in the Chinese Clinical Trial Registry (ChiCTR-IOR-17012174). If there is any change about the clinical research protocol or informed consent, the researchers will report the modifications to both the ethics committee and registry center timely. The Standard Protocol Items: Recommendations for Interventional Trials (SPIRIT) Checklist is given in Supplemental file 1.

### Patients and public involvement

None of the participants in the study participated in the design or evaluation. The results will be disseminated to study participants via email or phone messages.

## Trial status

This trial is currently recruiting participants. The recruitment began on Jan.1, 2018, and will be completed on Dec. 30, 2020.The protocol version number is 2016082601.

### Composition of the data monitoring committee (DMC)

The DMC of the study center comprises experienced experts on experimental statistics in the Guangzhou University of Traditional Chinese Medicine (GZUTCM). The DMC periodically reviews the trial on issues such as the execution of the trial, collection of the data, allocation concealment, and personal privacy protection. This committee will also provide advice on the modification or termination of the trial. The DMC is independent from the sponsor and has no competing interests.

### Public access to the protocol and data

In accordance with the data-sharing policy of CHICTR (http://www.chictr.org.cn), the data from this study will be uploaded to the ResMan database within 6 months after the trial is completed. The data will become available to outside researchers at the conclusion of the trial and following the publication of the main study findings as a limited-use dataset with documentation. The study participants will be informed about data sharing with external investigators in the consent forms. All outside investigators will be asked to sign a data-use agreement to protect study participant confidentiality.

## Discussion

This trial is a single-blind, randomized controlled study. Patients who meet the inclusion criteria will be recruited and randomly assigned to combined treatment group (JTN+MT) or JTN group.

JTN is a new school of acupuncture and moxibustion established by professor Jin Rui. Based on the theory of traditional Chinese medicine and previous relevant studies [31, 32], the points we mainly focus on were chosen to improve limb function, improve muscle strength, and reduce muscle spasm, which are necessary for promoting the restoration of upper limb function and improving the patients’ limb movement abilities and quality of life. The meridians we chose are the governor meridian, hands and feet three Yin and three Yang meridians, and tendons [11–13]^.^ The main points are temporal three needles, hand three needles, and clonic three needles of the upper limbs. Temporal three needles have been shown to significantly improve upper limb muscle strength, resulting in the rehabilitation of hemiplegic limbs after CIS [25]. Hand three needles have been shown to relieve upper limb tightness, paralysis, pain, sensory disturbance, and so on [7]. Clonic three needles of the upper limbs have been shown to relieve spasms [25]. These main points can adjust the upper limb meridians and help alleviate tendon stagnation, which would eventually promote the recovery of upper limb function.

MT originated in the 1990s. Ralnachandran [33] was the first person to use mirrors to treat patients with phantom limb pain after amputation in 1994. Subsequent studies have constantly applied MT in the rehabilitation of patients with stroke, hand trauma, and chronic regional pain syndrome [34]. MT involves many processes, such as motion observation, motor imagination, and imitation learning. MT uses the “illusion” to provide visual feedback to the brain of the “mistake,” which is that both hands are being controlled the same time, to activate the domination of hand movement neurons and promote brain function restructuring [16]. One study has further indicated that MT can promote the recovery of limb function after stroke, thereby improving the ability to perform ADL [35].

A related study [36] indicated that traditional acupuncture therapy combined with modern rehabilitation training can significantly promote the recovery of limb function in patients with CIS, especially the distal joint. In this study, JTN combined with MT activated relevant sensory areas in the brain through visual feedback to improve the overall motor function of patients. It was concluded that mirror neurons in the brain were directly involved in the imitation and understanding of movements under the action of image stimulation, and the brain-damaged areas of motor function in patients with paraplegia were reorganized to compensate for the loss of motor function.

The fMRI results before and after treatment will be used as the reference for clinical efficacy evaluation in this study to provide valuable information on the changes in neuronal activity in local brain areas caused by CIS and information on the node attributes in the functional connectivity group.

However, the efficacies of JTN and MT have not been systematically compared, and any possible synergistic effect of their combination has not been evaluated. Therefore, we propose a rigorous, randomized, controlled clinical trial to test whether the two treatment methods applied in combination are more effective than the single method. In this study, we will systematically evaluate the effects of different treatments on upper limb motor dysfunction after CIS and assess upper limb motor function and daily activity. The long-term stability in the improvements will also be evaluated during a 12-week follow-up examination after the intervention.

## Limitations of the study

One of the limitations of this study is that the study period is short, so the long-term effects may not be clear. A 12-week follow-up examination after the intervention will settle this problem, which will also provide a reference for further studies in the future. Another limitation is its non-double-blind design. However, the outcome assessors and statistical analysts will be blinded to the intervention to decrease possible bias and ensure the quality of this trial.

## Conclusion

In conclusion, the results of this study are expected to provide evidence on the efficacy and safety of JTN, to demonstrate and improve the clinical effect of JTN combined with MT in the treatment of upper limb dysfunction after CIS and to demonstrate the effect and safety of the therapy, thereby providing the scientific basis for its clinical promotion. Using fMRI brain functional imaging, the central brain mechanisms of these effects will be investigated, which will provide a strong evidence-based medical basis for further popularizing the application of JTN combined with MT.

## Supplementary Information

**Additional file 1.** Standard Protocol Items: Recommendations for Interventional Trials (SPIRIT) Checklist.

**Additional file 2.** Informed Consent Form (in Chinese).

## Data Availability

The datasets generated and/or analyzed during the current study and the full protocol will be available from the corresponding author on reasonable request after the study completion, or the records will be shared on the Chinese Clinical Trial Registry website (http://www.chictr.org.cn) within 6 months after the trial is completed.

## Abbreviations

CIS: Cerebral ischemic stroke
JTN: JIN’s three-needle acupuncture
MT: Mirror therapy
TCM: Traditional Chinese Medicine
fMRI: Functional magnetic resonance imaging
MVF: Mirror visual feedback
FMA-UE: Fugl–Meyer Assessment for Upper Extremity
MAS: Motor assessment scale
ARAT: Action research arm test
ADL: Activities of daily living
