# Supplementary material for: Using brain functional magnetic resonance imaging to evaluate the effectiveness of acupuncture combined with mirror therapy on upper limb function in patients with cerebral ischemic stroke: a study protocol for a randomized, controlled trial
Source: Trials. 2021 Jan 12;22:53. doi: 10.1186/s13063-020-04955-2 (PMC7805106; doi:10.1186/s13063-020-04955-2)
Supplement: Supplementary file 2 — Additional file 2. Informed Consent Form (in Chinese). [file 13063_2020_4955_MOESM2_ESM.pdf]

临床研究知情同意书

项目名称：针刺结合镜像疗法对脑梗死患者上肢功能及 fMRI 脑功能效应机制的单盲、随机对照试验

申办者：崔韶阳

临床试验机构：广州中医药大学深圳医院（福田）

版本号：V1.1

主研究者：崔韶阳

个人阅读材料

尊敬的患者，请您仔细阅读本文，欢迎您提出问题并与您的家人、亲属、朋友或我们讨论。

现邀请您参加本临床研究。研究目的是提高针刺结合镜像训练治疗脑卒中偏瘫患者上肢功能障碍的临床疗效，并对靳三针疗法作临床规范化研究，观察针刺结合镜像训练治疗脑卒中偏瘫患者上肢功能障碍的确切效果及其安全性，为其临床推广应用提供科学依据。

是否参加这项研究完全取决于您的意愿，在您做出决定之前请详细阅读此材料。它可以帮助您全面了解这项研究的目的、方法、研究过程，参加研究可能带来的益处和不便，以及您的权益。本知情同意书提供给您的信息可以帮助您决定是否参加此项临床试验，如有任何疑问请向负责该项目试验的研究者提问，也可与您的家人、亲属、朋友一起讨论，以确保您充分理解有关的内容。您是否参加本项试验是自愿的，如果同意参加该临床试验，请在知情同意书的声明中签字。

一. 这是一项什么样的研究？【研究背景和目的】

这项研究名称是针刺结合镜像疗法对脑梗死患者上肢功能及 fMRI 脑功能效应机制的单盲、随机对照试验。研究目的是提高针刺结合镜像训练治疗脑卒中偏瘫患者上肢功能障碍的临床疗效，并对靳三针疗法作临床规范化研究，观察针刺结合镜像训练治疗脑卒中偏瘫患者上肢功能障碍的确切效果及其安全性，为其临床推广应用提供科学依据。本研究已获得针刺结合镜像疗法对脑梗死患者上肢功能及 fMRI 脑功能效应机制的单盲、随机对照试验的立项，已通过广州中医药大学深圳医院（福田）伦理委员会的审查，认为本研究是遵从了国际公认的《赫尔辛基宣言》原则，符合医疗道德的。

二. 参加这项研究是自愿的吗？

参加本项研究是自愿的。您有权决定是否参加这项研究，不参加本研究不需要任何理由。不参加本研究不会遭到任何的歧视或报复，完全不影响您与医生的关系，也不影响您的医疗利益，您将继续得到您的医生给予的诊疗。

三. 哪些人不宜参加本项目研究

如果您有以下任何一项情况，将不宜参加本项研究：

(1) 医生已明确您不符合诊断标准和纳入标准；

(2) 妊娠或哺乳期妇女，及近 3 个月内有生育意向的妇女；

(3) 合并心、肝、肾、造血系统、内分泌系统等严重原发性疾病及感染性或传染性疾病、精神病患者；

(4) 有出血倾向或出血性疾病者；

(5) 有晕针以及晕血病史者；

(6) 拒绝签署知情同意书者。

四. 这项研究中使用的方案是什么？

治疗组：“靳三针”针刺治疗结合镜像治疗。

对照组：靳三针针刺治疗。

操作方法：用 30 号 1.5 寸不锈钢毫针，头部平刺进针 1 寸左右，四肢穴直刺进针常规深度，得气后留针 30 分钟，间隔 10 分钟捻针 1 次。平补平泻，阴阳偏盛者，随证施用补泻手法，1 次/日，5 次/周，1 个月为 1 个疗程。

五.如果参加研究将需要做什么?

您如果参加本研究，在签署知情同意书后，就进入筛选期， 如果医生认为您符合入选标准，且适合参加本项研究，将根据计算机产生的随机数字，决定您接受治疗的方式。您将会有 50%机会进入治疗组，50%机会进入对照组。这是一项单盲的研究，即您不知道您将会进入哪一个治疗组，但是在紧急情况下，是可以获知这一信息。

针刺治疗前后都需要对运动功能评价指标（上肢简化 Fugl-Meyer 评分，FMA）、运动功能评估量表（motor assessment scale，MAS）、上肢运动研究测试（the action research arm test，ARAT）、视觉模拟评分（visual analogue scale score，VAS）、日常生活活动能力（ADL、Barthel 指数）、中医临床症状、fMR 进行评分。次要观察指标主要包括患者血常规、尿常规、便常规、肝肾功能，以判断您接受的治疗是否安全和有效。

观察期间请您按照医嘱返院治疗，医生将详细告诉您治疗时间、方法、复诊时间，在此期间您不能自行服用其他药物或接受其他治疗，如果需要请先咨询您的医生。

六.相关费用：在本次研究中，我们将为您提供免费的针刺治疗及相应的血液指标检测，如果您同时合并其他疾病，且与本次研究无关，其所需的治疗和检查，

以及因研究中止改用其他治疗措施的费用，将不在免费的范围之内。如果经正规评定确认确实出现了本研究造成您人身损害的情况，课题研究组将支付您相应的医疗费用和根据相关法律法规进行补偿。

七．参加研究可能的受益

您和社会将可能从本项研究中受益，也可能无直接受益。此种受益包括您的病情有可能获得改善,以及本项研究可能帮助进一步提高针刺结合镜像训练治疗脑卒中偏瘫患者上肢功能障碍的临床疗效，并对靳三针疗法作临床规范化研究，观察针刺结合镜像训练治疗脑卒中偏瘫患者上肢功能障碍的确切效果及其安全性，为其临床推广应用提供科学依据，有益于与您有类似病情患者的治疗。

八．参加研究可能的不良反应、风险及不便

1.在治疗过程中，您有可能出现如下不良反应：

（1）晕针：在针刺过程中突然发生头晕、目眩、心慌、恶心，甚至晕厥的现象。 晕针常由于体质虚弱、精神紧张；或饥饿、大汗、大泻、大出血之后；或体位不当等，以致针刺时或留针时发生此证。

（2）滞针：进针后或行针过程中，于提插捻转或出针时，针下感觉非常沉重、紧涩，甚至捻转不动，进退困难者，同时相应针刺部位感到异常疼痛的现象。

（3）断针：因针具质量欠佳，针身或针根有剥蚀损坏，针刺时针身全部刺入或行针时强力捻转提插， 或因肌肉强烈收缩及患者改变体位，滞针和弯针现象未及时正确处理，导致针身折断，残端留在患者体内。本研究选用正规厂家生产针具，并研究者经过专业培训操作规范，可最大限度降低断针发生几率。

（4）感染：一般情况下针刺的针孔微小,同时机体有一定的抵抗力，一般不容易引起感染，然而存在少数患者因抵抗力低下出现针刺感染的可能。本研究操

作者经过规范培训，在针刺治疗时会进行常规消毒，尽可能最大限度减少针刺感染几率。

2.可能存在的风险及不便：

（1）在本研究的整个治疗过程中受试者不能接受其他相关治疗或自行服用药物，如需服用药物或接受其他治疗必须咨询主管医生，由此可能对您造成一定不便；如果受试者在未告知主管医生的情况下接受了会影响治疗结果的相关治疗或服用可造成影响的药物，将有可能被从试验中排除，由此造成的不良后果将由受试者个人承担。

（2）为避免对治疗效果造成影响，受试者必须按照医嘱及时返院接受治疗，由此可能造成一定日程安排及交通上的不便。

（3）凡入组后发现严重躯体疾病，或出现病情不可预见的急剧恶化，或出现严重不良反应者，为避免延误病情及确保受试者安全，将从试验中脱出，由此可能造成治疗过程不完整或治疗效果受到影响。

（4）所有治疗都可能产生不良反应，如果在研究中您出现任何不适，或病情发生新的变化，或任何意外情况，不管是否与针刺治疗有关，均应及时通知您的医生，他/她将对此作出判断和医疗处理。

九．如何保护您的隐私权？

您的医疗记录（包括研究病历及理化检查报告等）将按规定保存在医院。您参加研究及在研究中的个人资料均属保密，研究结束后的研究结果报告也不会显露您的个人身份。国家食品药品监督管理部门、伦理委员会、研究者和申办者代表将被允许查阅您的医疗记录，以便核查临床研究的程序和/或数据。我们将在现有的法律范围内，尽一切努力保护您个人医疗资料的隐私。

十．重要提示

如果您是妊娠期或哺乳期妇女，则不宜参加本研究。育龄妇女或配偶为育龄期女性，应在研究期间采取 1 种及以上方式避孕措施，避免受孕，若受孕则中止临床研究，并马上联系您的医生采取相应措施。

为了保证临床研究的可靠性，在整个临床研究过程中，希望您能做到以下几点：①不自行使用其他相关治疗药物；②研究过程中，有任何不适，请及时向您的主管医生反映。

本研究的治疗方案并不是您目前所患疾病的唯一治疗选择，您可以和医生商量后，再决定是否参加本研究。

十一．参加研究后可以退出吗？

是否参加完全取决于您的自愿。您可以拒绝参加此项研究，或在研究过程的任何时间退出研究，不需要任何理由，这都不会影响您和医生的关系，都不会影响对您的医疗或其他方面利益的损失，您不会遭到任何歧视或报复。

您的医生处于对您的最大利益考虑，可能会随时中止您参加本项研究。

如果您不参加本项研究，或中途退出，还有很多其他替代治疗措施。您不必为了治疗您的疾病而必须参加本项研究。如果您因为任何原因从研究中退出，出于对您的最大利益考虑，您可能被询问有关您使用药物情况，如果医生认为需要，您也可能被要求进行实验室检查和体格检查。

如果您通过充分考虑之后选择参加本研究，我们希望您能够坚持完成全部研究过程。

十二．更多信息的获取

您可以在任何时间提出有关本项研究的任何问题，您的医生将会给您留下联

系方式以便能回答您的问题。如果在研究过程中有任何重要的新信息，可能影响您继续参加研究的意愿时，您的医生将会及时通知您。

十三．现在该做什么?

是否参加本项研究由您自己决定。您也可以和您的家人讨论后再做出决定。在您做出参加研究的决定前，请尽可能向您的医生询问有关问题，直至您对本项研究完全理解。

最后，感谢您阅读以上材料。如果您决定参加本项研究，请告诉您的医生，他们会为您安排一切有关研究的事务。请您保留这份资料。如您对本研究中您的权益有任何疑问，可联系本中心伦理委员会，联系电话：020-36591965.。

受试者声明

我已经仔细阅读了本知情同意书, 我有机会提问而且所有问题均已得到解答。我理解参加本项研究是自愿的, 我可以选择不参加本项研究, 或者在任何时候通知研究者后退出而不会遭到歧视或报复, 我的任何医疗待遇与权益不会因此而受到影响。

如果我需要其他诊断/治疗, 或者我没有遵守试验计划, 或者有其他合理原因, 研究者可以终止我继续参与本项临床研究。

我自愿同意参加该项临床研究, 我将收到一份签过字的"知情同意书"副本。

受试者签名: 日期: 年 月 日

联系电话:

法定代理人签字【如适用】:

同受试者关系: 日期: 年 月 日

研究者声明

我已准确地将知情同意书内容告知受试者并对受试者的提问进行了解答, 受试者自愿参加本项临床研究。

研究者签名:

联系电话: 日期: 年 月 日
